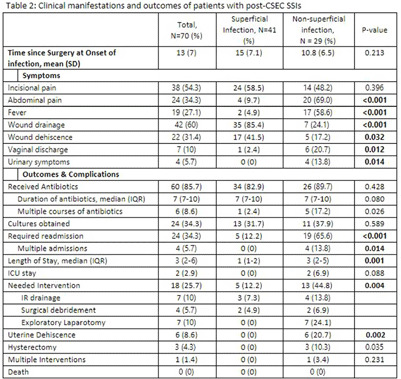# A Deep Dive into Post-Cesarean Section Surgical Site Infections

**DOI:** 10.1017/ash.2025.413

**Published:** 2025-09-24

**Authors:** Rija Alvi, Clare Shanahan, Abigail Ruby, Eman Chami, Geehan Suleyman

**Affiliations:** 1 Henry Ford Hospital; 2 Henry Ford Health System; 3 Henry Ford Health System; 4 Henry Ford Health

## Abstract

Surgical site infection (SSI) is one of the most common complications following cesarean section (CSEC) and adds significant burden to the healthcare system. We aimed to explore factors associated with increased risk of these infections and to compare disease severity based on tissue level.

Observational study of post-CSEC SSIs from Jan 2021-Dec 2023 at Henry Ford Hospital in Detroit. SSIs were defined according to National Healthcare Safety Network (NHSN) criteria. Cases were categorized as superficial incisional (SI), deep incisional (DI) and organ space (OS). Demographics, risk factors, clinical features and outcomes were evaluated. DI and OS were grouped together into non-superficial infections for comparative analysis. 70 (3%) of 2,230 CSECs performed during the study period met post-CSEC SSI criteria, of which 41 (60%) were SI, 4 (6%) DI, and 25 (34%) OS (Table 1). Majority of patients were black (51.5%) with BMI>30 (56%), had Medicaid insurance (66%) and underwent emergent CSEC (60%). Anemia (hemoglobin 3 manual exams prior to surgery (55% vs. 29%, p=0.017) were significantly more common among patients with non-superficial infections. Receipt of perioperative antibiotics was similar between the two groups, and most were administered within 1 hour of incision; cefazolin was frequently used. Incisional pain and drainage were the most prevalent symptoms (Table 2). Abdominal pain (69% vs. 10%, 3 manual exams and worse clinical outcomes compared to patients with superficial infections. Implementing evidence-based practices and recommendations are therefore critical to reduce the morbidity associated with non-superficial CSEC infections.

**Figure:**
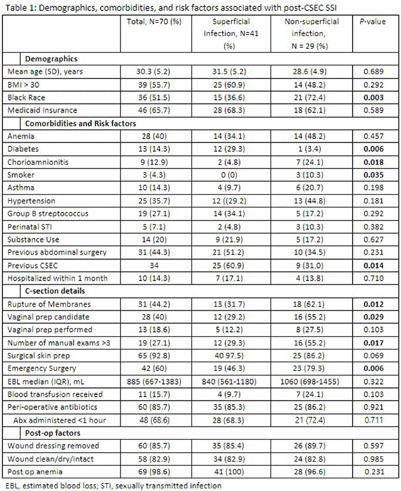

**Figure:**